# Development and validation of a nomogram to predict overall survival in patients with redefined anaplastic thyroid carcinoma based on the SEER database

**DOI:** 10.1007/s10147-024-02495-2

**Published:** 2024-04-07

**Authors:** Chuyue Zhang, Bin Li, Yan Yang

**Affiliations:** Department of General Surgery, the 920th Hospital of Joint Logistics Support Force, PLA, Kunming, Yunnan People’s Republic of China

**Keywords:** Thyroid carcinoma, Anaplastic, Primary squamous cell carcinoma of thyroid, Prognosis, Risk, Nomogram, SEER program

## Abstract

**Background:**

According to the latest classification of thyroid tumors released by the WHO in 2022, primary squamous cell carcinoma of the thyroid (PSCCTh) is classified as anaplastic thyroid carcinoma (ATC). The objective of this study was to determine the differences in characteristics between ATC and PSCCTh and develop a nomogram to predict overall survival patients with the redefined anaplastic thyroid carcinoma (rATC).

**Methods:**

Patients diagnosed with ATC and PSCCTh between 2000 and 2018 from the Surveillance, Epidemiology, and End Results (SEER) database were enrolled and randomly divided into a training cohort and a validation cohort with a ratio of 7:3. Overall survival (OS) and cancer-specific survival (CSS) was estimated using the Kaplan–Meier method and compared using log-rank tests. The univariate and multivariate Cox proportional hazards regression analyses were used to determine independent prognostic factors of rATC patients. We then developed and validated nomograms to predict the 3-, 6- and 12-month OS of rATC and the results were evaluated by C-index and calibration curves.

**Results:**

After application of the inclusion and exclusion criteria, a total of 1338 ATC and 127 PSCCTh patients were included in the study. Further, OS and CSS of patients with PSCCTh were better than that of patients with ATC. Prognostic factors were not identical for the two cancers. Multivariate Cox model analysis indicated that age, tumor size, metastasis, surgery, radiotherapy, chemotherapy are independent prognostic factors for CSS in patients with ATC; while for patients with PSCCTh, the corresponding factors are age, and surgery. We selected six survival predictors (age, tumor size, metastasis, surgery, radiation, and, chemotherapy) for nomogram construction. The C-indexes in the training and validation cohort were 0.740 and 0.778, respectively, reflecting the good discrimination ability of the model. The calibration curves also showed good consistency in the probability of 3-, 6-, and 12-month OS between the actual observation and the nomogram prediction.

**Conclusion:**

We constructed a nomogram to provide a convenient and reliable tool for predicting OS in rATC patients. Prognostic factors influencing CSS were not identical in patients with ATC and PSCCTh. These findings indicate that different clinical treatment and management plans are required for patients with these two types of thyroid cancer.

Anaplastic thyroid cancer (ATC) is a follicular-derived tumor of the thyroid gland. It is characterized by strong invasiveness, rapid disease progression, high metastasis rate, and poor prognosis [1]. It accounts for approximately 1%–2% of all primary thyroid cancers and is more commonly found in elderly individuals [2]. Giant cells, spindle cells, and squamous cells show obvious pleomorphism, while anaplastic tumors have no clear differentiation [3]. Primary squamous cell carcinoma of thyroid (PSCCTh) is an extremely rare and highly invasive malignant tumor, accounting for less than 1% of all primary thyroid cancers [2]. The prognosis is extremely poor. In terms of cytology, PSCCTh consists of large pleiomorphic epithelial cells, with frequent keratinization and necrotic components [3]. The clinical symptoms of ATC and PSCCTh are similar, mainly characterized by neck masses and enlargement of neck lymph nodes. Due to its invasiveness, the tumor grows at an extremely fast rate and can cause symptoms of compression within a short period of time, such as difficulty breathing, difficulty swallowing, hoarseness, and neck pain [4]. Both ATC and PSCCTh present with abundant squamous cell-like cells; hence, the two conditions are easy to confuse during clinical diagnosis and are treated similarly.

According to the new fifth edition of the *WHO Classification of Endocrine and Neuroendocrine Tumors* that relate to the thyroid gland, PSCCTh is now classified as a morphologic pattern of ATC [3, 5]. Currently, there is no research that has integrated the clinical and pathological information of patients with these two cancers and develop a nomogram to predict overall survival patients with the redefined anaplastic thyroid carcinoma (rATC). The similarities and differences between ATC and PSCCTh have become a focus of increased research attention. Both types of primary thyroid cancer are highly aggressive and have poor prognosis. At present, combined treatments, including surgery, radiotherapy, and chemotherapy, are often used in the clinic [6, 7]; however, the role of adjuvant therapy for these two cancers remains unclear. In addition, differences in overall survival (OS) and cancer-specific survival (CSS) between patients with ATC and PSCCTh have not been extensively studied. Given the rarity of these two diseases, conducting prospective studies with large samples is very difficult. This study conducted a retrospective analysis using the data extracted from the Surveillance, Epidemiology, and End Results (SEER) database of the National Cancer Institute in the United States.

## Methods

### Patients and selection criteria

A retrospective cohort study was conducted using the SEER program of the National Cancer Institute (NCI). We obtained permission to access the data files from the SEER program by NCI with the reference number 21091—November 2021. All the information for patients with ATC and PSCCTh was extracted from the SEER database by the SEER*Stat program (version 8.4.1.2). The extraction conditions were as follows: ‘‘the location of the disease: thyroid, the ICD-9 site codes C73.9.’’ and ‘‘diagnosis year: 2000–2018.’’ The following variables were extracted: patient ID, age at diagnosis, sex, race recode, marital status at diagnosis, laterality, tumor size summary, radiation recode, chemotherapy recode, ICD-O-3 Hist/behav, survival months, and vital status recode (study cutoff used). The inclusion criteria were as follows: (1) confirmed pathology of primary ATC and PSCCTh; and (2) all cases assigned the ICD-O-3 histology coded as 8012/3/, 8020/3, 8021/3, 8030/3–8032/3, ATC and 8070/3–8076/3, PSCCTh. The exclusion criteria were as follows: (1) combination with other malignant tumors; (2) diagnoses made at autopsy or noted on the death certificate only; and (3) incomplete information. As patient data identified from the database were deidentified and available to the public for research purposes, the ethical approval of the present study was waived by the local ethics committee.

### Statistical analysis

In the present study, all statistical analysis was performed with R software (version 4.2.2), and a *P* value < 0.05 (two side) was considered as statistical significance. In the SEER database, OS is calculated as the number of months from cancer diagnosis to the date of death. For the estimation of CSS, patients who died from causes other than thyroid carcinoma of the thyroid were censored. The Kaplan–Meier method was used to evaluate patient OS and CSS, and the log-rank method was used to assess differences in OS and CSS between patients with the two types of thyroid cancer. Single-factor and multi-factor Cox regression models were used to assess the impact of demographic and clinical factors on CSS in patients with ATC and PSCCTh; variables with *P* < 0.05 on univariate test were included in the multivariate cohort.

All of the eligible rATC cases were randomly divided into either the training or validation cohort (the split ratio was 7:3), with the training cohort being used to establish the predictive model and to construct the nomogram and risk classification system. Validation of the model was carried out using the data from the validation cohort. First, a univariate Cox proportional hazards model was used to check each parameter’s power in predicting OS. Second, factors with a *P* < 0.05 in univariate analysis were further analyzed in a multivariate Cox proportional hazards model using a backwards model selection procedure. Finally, factors that were included in the final model were utilized to build the nomogram.

According to the regression coefficients of each factor in the multivariate analysis, the predictive model was virtualized by the nomogram. The validation of the nomogram was performed using the concordance index (C-index), and, calibration curves. The C-index was used to reflect the predictive accuracy and discrimination ability of each factor and of the nomogram. Calibration curves (1000 bootstrap resamples) were generated to test the calibration of the nomogram.

Calibration curves predicting the 3-, 6-, and 12-months OS of rATC patients in the training cohort (A, B, C) and the validation cohort (D, E, F). The x-axis indicates the predicted survival probability, and the y-axis indicates the actual survival probability. The 45-degree line (gray line) indicates that the prediction agrees with actuality.

## Results

### Differences in survival time between patients with ATC and PSCCTh

**Fig. 1:**
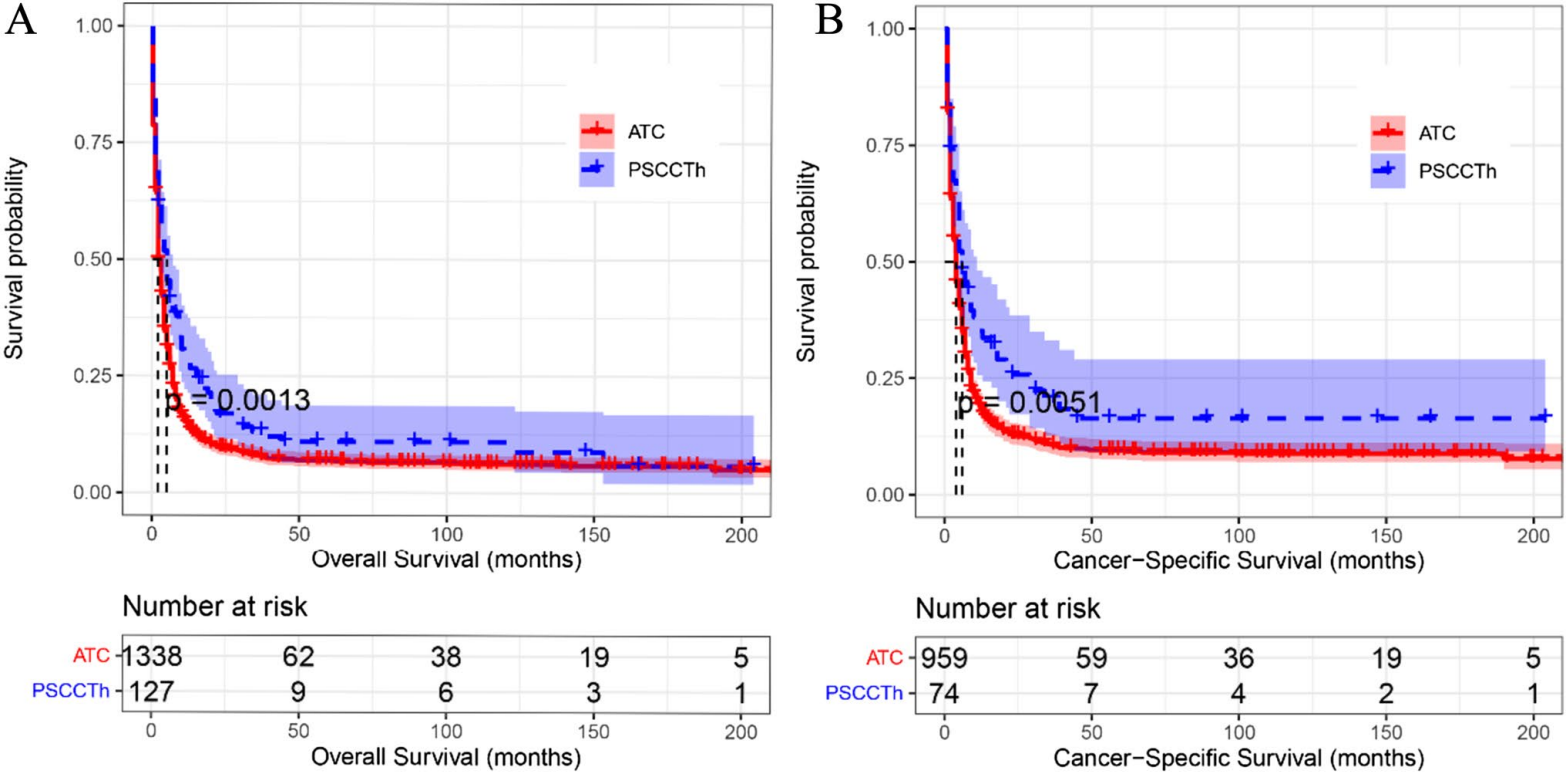
Kaplan–Meier curves of OS and CSS in patients with ATC and PSCCTh

To evaluate the OS and CSS prognosis of patients with ATC and PSCCTh, we visualized and analyzed OS and CSS in patients from these two groups. Kaplan–Meier curves indicated that the median OS of patients with ATC was 2 months, while that of patients with PSCCTh was almost 5 months. Log-rank test analysis demonstrated that OS was longer in patients with PSCCTh than in those with ATC (*P* < 0.01) (Fig. 1a). The median CSS of patients with ATC was 4 months, while that of patients with PSCCTh was almost 6 months. Log-rank test analysis demonstrated that CSS was longer in patients with PSCCTh than in those with ATC (*P* < 0.01) (Fig. 1b). The median OS of rATC patients was 3 months, and the 3-, 6-, and 12-month OS rates were 43.7%, 28.2%, and 16.0%, respectively. The median CSS of rATC patients was 4 months, and the 3-, 6-, and 12-month CSS rates were 55.9%, 36.1%, and 20.2%, respectively. The choice of surgical method is the common independent influencing factor of CSS in ATC and PSCCTh patients.

### Patient baseline characteristics

A total of 1465 patients with rATC were extracted from the SEER database from 2000 to 2018. Among these patients, 1338 (91.33%) had ATC, 127 (8.67%) had PSCCTh. 573 (39.11%) patients were male, 892 (60.89%) patients were female. The age of ATC patients was (69.20 ± 12.62) years, PSCCTh patients was (70.73 ± 12.53) years. A total of 637 patients (43.48%) received surgical treatment, including 297 cases (20.27%) of subtotal or near-total thyroidectomy and 340 cases (23.21%) of total thyroidectomy. A total of 1033 patients died of thyroid cancer, including 959 cases (92.84%) of ATC and 74 cases (7.16%) of PSCCTh. The demographics and clinicopathological characteristics of the training cohort and validation cohort are presented in Table 1.

**Table 1:** Demographic and clinicopathological characteristics of 1,033 ATC and PSCCTh patients who died due to thyroid cancer, as well as 1,465 rATC patients [*n* (%)]

Characteristic	ATC (*n* = 959)	PSCCTh (*n* = 74)	*P*	Training cohort (*n* = 1025)	Validation cohort (*n* = 440)	*P*
Age (year)			0.794			0.621
<50	68 (7.091)	4 (5.405)		56 (5.463)	31 (7.045)	
50–59	185 (19.291)	12 (16.216)		179 (17.463)	69 (15.682)	
60–69	270 (28.154)	22 (29.730)		263 (25.659)	116 (26.364)	
70–79	245 (25.547)	23 (31.081)		272 (26.537)	123 (27.955)	
≥ 80	191 (19.917)	13 (17.568)		255 (24.878)	101 (22.955)	
Sex			0.586			0.060
Male	371 (38.686)	31 (41.892)		417 (40.683)	156 (35.455)	
Female	588 (61.314)	43 (58.108)		608 (59.317)	284 (64.545)	
Ethnicity			0.200			0.915
White	761 (79.353)	55 (74.324)		797 (77.756)	348 (79.091)	
Black	78 (8.133)	4 (5.405)		88 (8.585)	38 (8.636)	
Asia–Pacific	110 (11.470)	13 (17.568)		127 (12.390)	49 (11.136)	
Other/Unknown	10 (1.043)	2 (2.703)		13 (1.268)	5 (1.136)	
Marital status			0.473			0.176
Single	124 (12.930)	6 (8.108)		127 (12.390)	55 (12.500)	
Married	546 (56.934)	44 (59.459)		558 (54.439)	252 (57.273)	
Divorced	66 (6.882)	6 (8.108)		68 (6.634)	28 (6.364)	
Widowed	189 (19.708)	13 (17.568)		240 (23.415)	83 (18.864)	
Unknown	34 (3.545)	5 (6.757)		32 (3.122)	22 (5.000)	
Tumor site			0.578			0.860
Bilateral	4 (0.417)	0 (0.000)		4 (0.390)	2 (0.455)	
Unilateral	955 (99.583)	74 (100.000)		1021 (99.610)	438 (99.545)	
Tumor size (cm)			0.155			0.129
≤ 2.0	26 (2.711)	4 (5.405)		27 (2.634)	11 (2.500)	
2.1–4.0	100 (10.428)	7 (9.459)		87 (8.488)	55 (12.500)	
> 4.0	559 (58.290)	35 (47.297)		568 (55.415)	233 (52.955)	
Unknown	274 (28.571)	28 (37.838)		343 (33.463)	141 (32.045)	
Metastasis			0.830			0.198
Localized	75 (7.821)	4 (5.405)		86 (8.390)	24 (5.455)	
Regional	230 (23.983)	20 (27.027)		233 (22.732)	94 (21.364)	
Distant	633 (66.006)	48 (64.865)		682 (66.537)	312 (70.909)	
Unknown	21 (2.190)	2 (2.703)		24 (2.341)	10 (2.273)	
Surgery			0.339			0.129
Non-surgical Therapy	476 (49.635)	43 (58.108)		582 (56.780)	246 (55.909)	
Subtotal/Near-total Thyroidectomy	208 (21.689)	12 (16.216)		195 (19.024)	102 (23.182)	
Total thyroidectomy	275 (28.676)	19 (25.676)		248 (24.195)	92 (20.909)	
Radiation			0.193			0.335
Yes	629 (65.589)	43 (58.108)		552 (53.854)	249 (56.591)	
Refused/unknown	330 (34.411)	31 (41.892)		473 (46.146)	191 (43.409)	
Chemotherapy			0.383			0.322
Yes	504 (52.555)	35 (47.297)		428 (41.756)	196 (44.545)	
No/unknown	455 (47.445)	39 (52.703)		597 (58.244)	244 (55.455)	
Histopathology			**–**			0.236
ATC	**–**	**–**		942 (91.902)	396 (90.000)	
PSCCTh	**–**	**–**		83 (8.098)	44 (10.000)	

### Analysis of prognostic factors for CSS in patients with ATC and PSCCTh

**Fig. 2:**
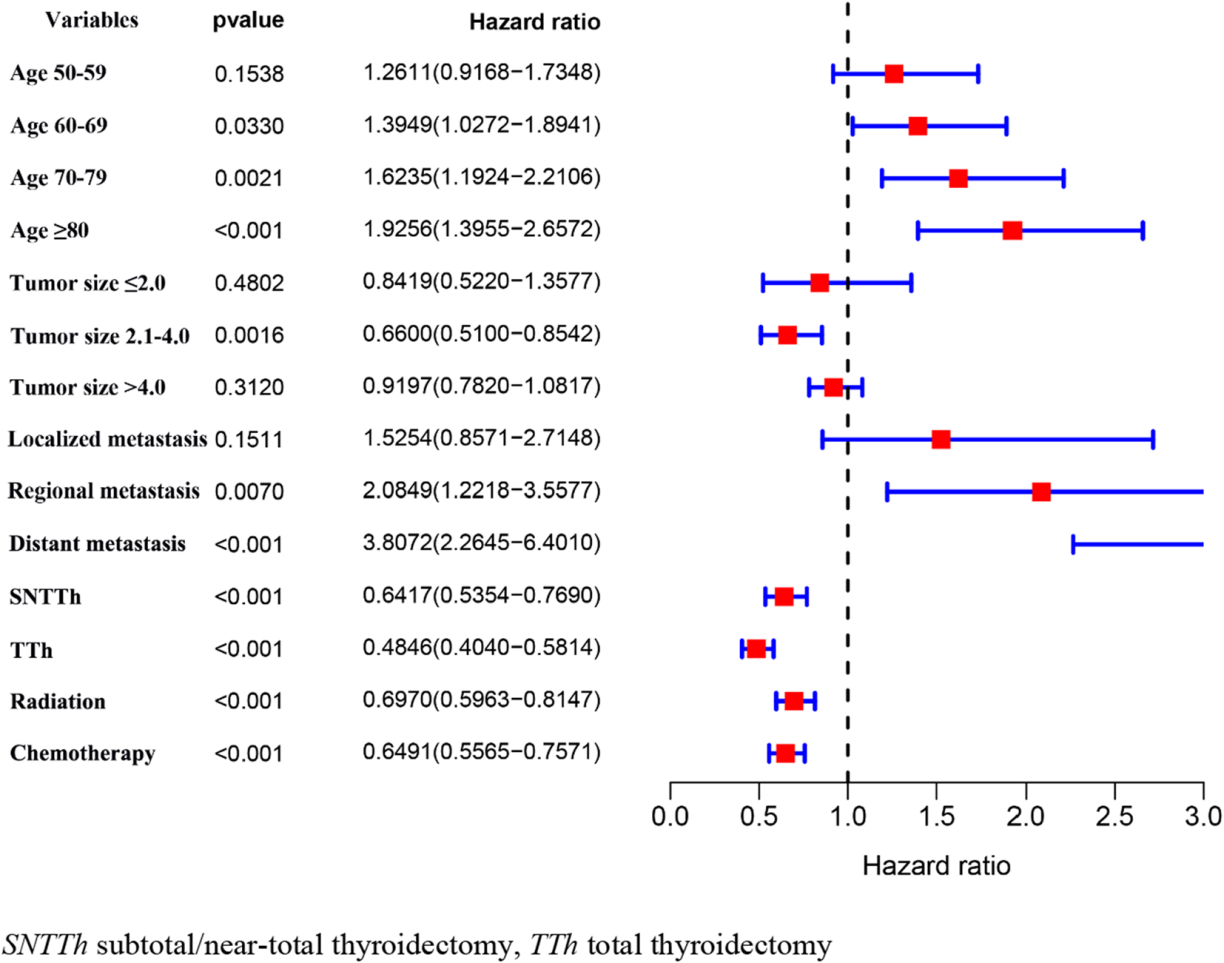
Multivariate Cox regression model was used to analyze the forest plot of related factors of CSS in ATC patients

**Fig. 3:**
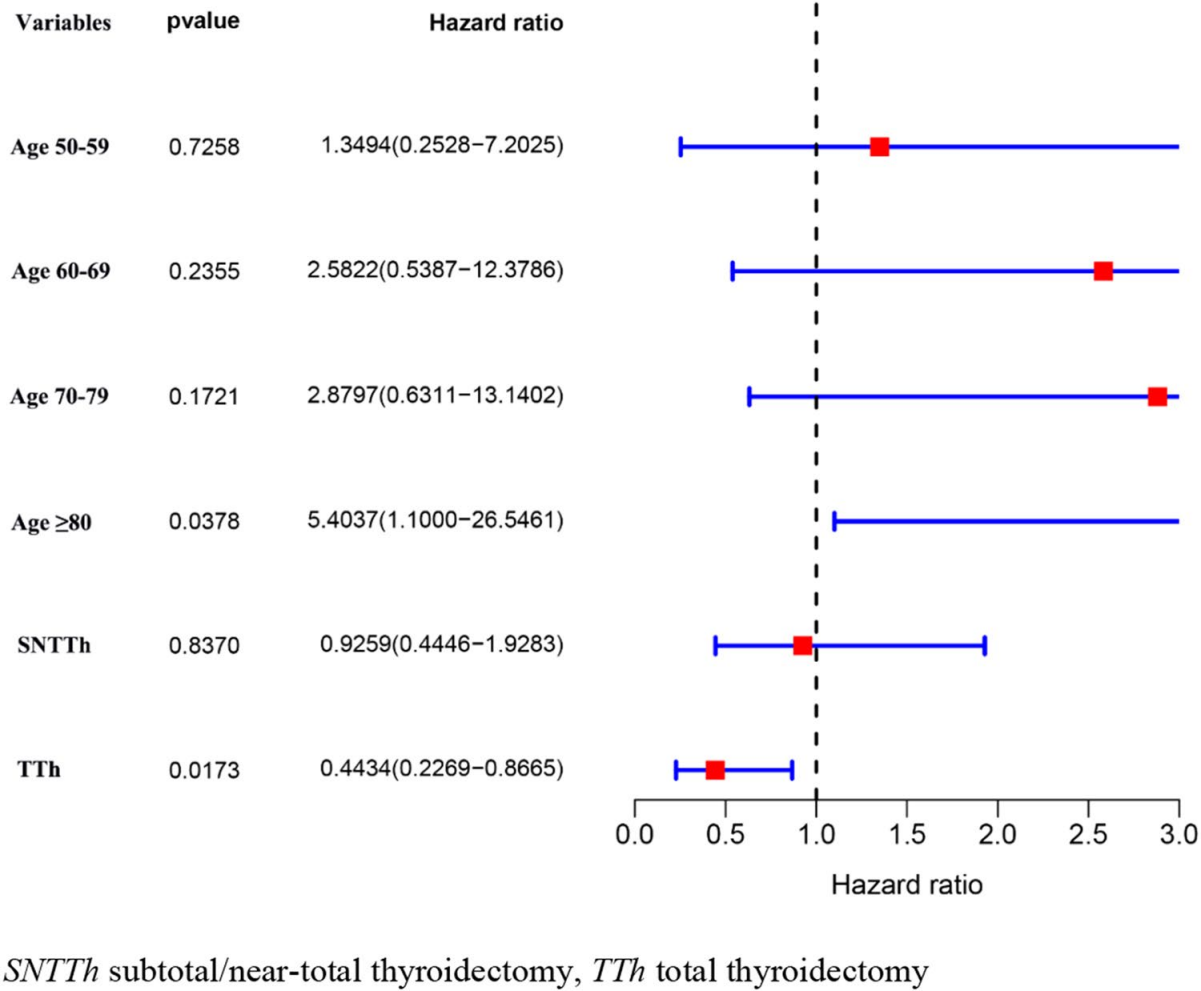
Multivariate Cox regression model was used to analyze the forest plot of related factors of CSS in PSCCTh patients

**Fig. 4:**
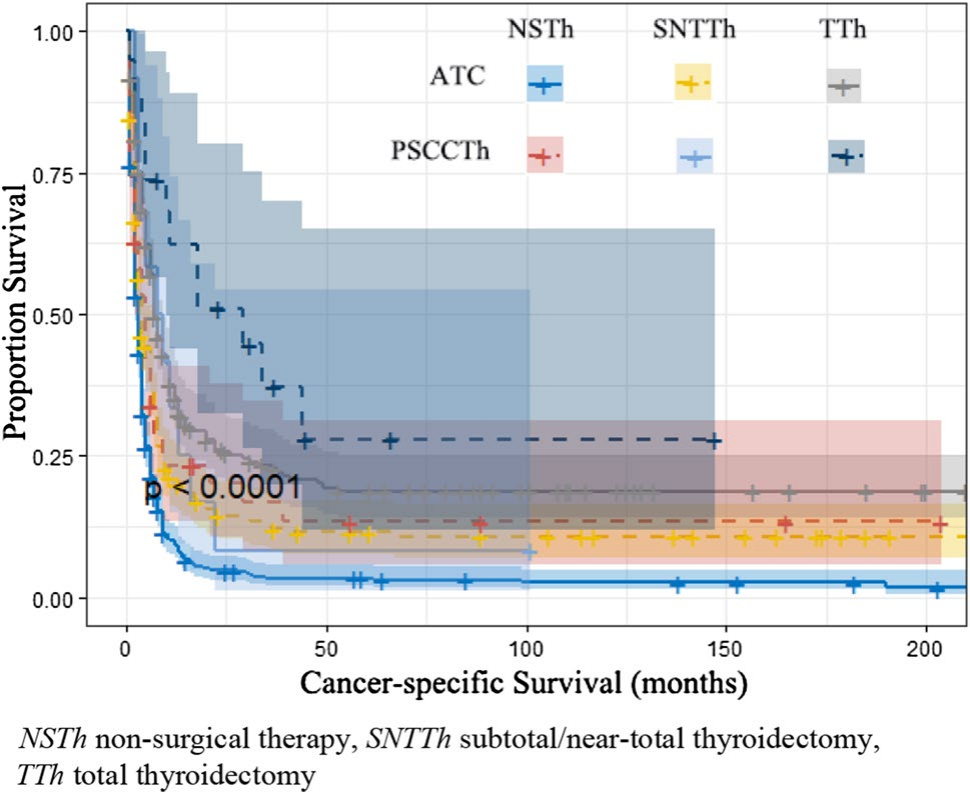
Kaplan–Meier curves of CSS in ATC and PSCCTh patients affected by surgical approach

To study influences on CSS in patients with ATC, we conducted univariate and multivariate Cox model analyses of various potential prognostic factors. The univariate analysis indicated that age, tumor size, metastasis, surgery, radiation, and, chemotherapy were significantly associated with CSS (*P* < 0.05). Based on the univariate analysis, the following six independent risk factors were selected for the multivariate analysis using a Cox regression: age (60–69 years: HR 1.395; 95% CI 1.027–1.894; *P* = 0.033 and 70–79 years: HR 1.624; 95% CI 1.192–2.211; *P* = 0.002 and ≥ 80 years: HR 1.926; 95% CI 1.396–2.657; *P* < 0.001), tumor size (2.1–4.0: HR 0.660; 95% CI 0.510–0.854; *P* = 0.002), metastasis (regional metastasis: HR 2.085; 95% CI 1.222–3.558; *P* = 0.007 and distant metastasis: HR 3.807; 95% CI 2.265–6.401; *P* < 0.001), surgery (SNTTH: HR 0.642; 95% CI 0.535–0.769; *P* < 0.001 and TTh: HR 0.485; 95% CI 0.404–0.581; *P* < 0.001), radiation (yes: HR 0.697; 95% CI 0.596–0.815; *P* < 0.001), chemotherapy (yes: HR 0.649; 95% CI 0.557–0.757; *P* < 0.001). The results of the univariate and multivariate analyses are presented in Table 2 and Fig. 2. To study influences on CSS in patients with PSCCTh, we conducted univariate and multivariate Cox model analyses of various potential prognostic factors. Univariate Cox analysis found that age and surgery were factors influencing CSS in patients with PSCCTh. Based on the univariate analysis, the following two independent risk factors were selected for the multivariate analysis using a Cox regression: age (≥ 80 years: HR 5.404; 95% CI 1.100–26.546; *P* = 0.038), surgery (TTh HR 0.443; 95% CI 0.227–0.867; *P* = 0.017). The results of the univariate and multivariate analyses are presented in Table 2 and Fig. 3. The CSS of patients undergoing total thyroidectomy is higher than that of patients undergoing subtotal thyroidectomy/near-total thyroidectomy. Same line total thyroidectomy, PSCCTh CSS is higher than the ATC patients (*P* < 0.001) (Table 3; Fig. 4).

**Table 2:** Univariate Cox regression model of factors associated with CSS in patients with ATC and PSCCTh

Characteristic	ATC		PSCCTh	
	HR (95% CI)	*P*	HR (95% CI)	*P*
Age (year)				
<50	1.000		1.000	
50–59	1.189 (0.867–1.631)	0.284	1.199 (0.249–5.781)	0.821
60–69	1.512 (1.117–2.046)	0.007	2.676 (0.611–11.721)	0.191
70–79	1.635 (1.206–2.215)	0.002	2.938 (0.683–12.628)	0.148
≥ 80	2.408 (1.764–3.287)	< 0.001	5.095 (1.116–23.256)	0.036
Sex				
Male	1.000		1.000	
Female	1.055 (0.917–1.214)	0.451	1.323 (0.781–2.243)	0.298
Ethnicity				
White	1.000		1.000	
Black	1.248 (0.975–1.597)	0.079	0.499 (0.121–2.064)	0.337
Asia–Pacific	1.161 (0.937–1.437)	0.172	14.770 (0.771–2.829)	0.240
Other/unknown	0.774 (0.347–1.730)	0.533	0.000 (0.000–Inf)	0.996
Marital status				
Single	1.000		1.000	
Married	0.890 (0.719–1.101)	0.284	0.622 (0.242–1.594)	0.322
Divorced	1.057 (0.764–1.463)	0.737	0.595 (0.142–2.499)	0.479
Widowed	1.245 (0.976–1.589)	0.078	1.658 (0.590–4.662)	0.338
Unknown	0.837 (0.544–1.286)	0.416	0.406 (0.097–1.704)	0.218
Tumor site				
Bilateral	1.000		**–**	
Unilateral	0.574 (0.185–1.784)	0.337	**–**	**–**
Tumor size (cm)				
Unknown	1.000		1.000	
≤ 2.0	0.601 (0.377–0.958)	0.032	1.845 (0.456–7.460)	0.390
2.1–4.0	0.703 (0.547–0.904)	0.006	1.256 (0.380–4.152)	0.709
> 4.0	0.853 (0.732–0.996)	0.044	1.615 (0.484–5.388)	0.436
Metastasis				
Unknown	1.000		1.000	
Localized	0.830 (0.475–1.452)	0.514	2.175 (0.284–16.670)	0.455
Regional	0.998 (0.597–1.667)	0.993	4.860 (0.668–35.380)	0.119
Distant	2.020 (1.227–3.323)	0.006	1.397 (0.087–22.450)	0.813
Surgery				
Non-surgical Therapy	1.000		1.000	
Subtotal/Near-total Thyroidectomy	0.633 (0.531–0.755)	< 0.001	0.848 (0.429–1.674)	0.635
Total Thyroidectomy	0.409 (0.345–0.485)	< 0.001	0.416 (0.214–0.807)	0.009
Radiation				
Refused/unknown	1.000		1.000	
Yes	0.630 (0.546–0.728)	< 0.001	1.071 (0.630–1.822)	0.799
Chemotherapy				
No/unknown	1.000		1.000	
Yes	0.581 (0.507–0.667)	< 0.001	1.273 (0.759–2.135)	0.361

*HR,* hazard ratio; *CI,* confidence interval

**Table 3:** The number of cases in which ATC and PSCCTh patients undergo various types of surgeries (*n* = 1033)

Characteristic	NSTh	SNTTh	TTh	Total
ATC	476	208	275	959
PSCCTh	43	12	19	74
Total	519	220	294	1033

### Independent risk factors in the training cohort

The univariate analysis indicated that age, marital status, tumor size, metastasis, surgery, radiation, chemotherapy, and histopathology were significantly associated with OS (*P* < 0.05). In contrast, sex, ethnicity, tumor site were not significant (*P* > 0.05). Based on the univariate analysis, the following eight independent risk factors were selected for the multivariate analysis using a Cox regression, the result of multiple regression analysis: age (70–90 year: HR 1.643; 95% CI 1.173–2.302; *P* = 0.004 and ≥ 80 year: HR 2.022; 95% CI 1.417–2.887; *P* < 0.001), tumor size (2.1–4.0: HR 0.606; 95% CI 0.466–0.787; *P* < 0.001), metastasis (distant metastasis: HR 2.037; 95% CI 1.575–2.634; *P* < 0.001), surgery (SNTTh: HR 0.573; 95% CI 0.479–0.686; *P* < 0.001 and TTh HR 0.453; 95% CI 0.380–0.540; *P* < 0.001), radiation (yes: HR 0.647; 95% CI 0.558–0.751; *P* < 0.001), chemotherapy (yes: HR 0.581; 95% CI 0.498–0.678; *P* < 0.001), histopathology (PSCCTh: HR 0.546; 95% CI 0.425–0.701; *P* < 0.001). The results of the univariate and multivariate analyses are presented in Table 4.

**Table 4:** Univariate and multivariate Cox analyses of overall survival for rATC patients in the training cohort (*n* = 1025)

Characteristic	Univariate Cox analysis		Multivariate Cox analysis	
	HR (95% CI)	*P*	HR (95% CI)	*P*
Age (year)				
< 50	1.000		1.000	
50–59	1.201 (0.852–1.692)	0.296	1.093 (0.773–1.544)	0.616
60–69	1.550 (1.114–2.156)	0.009	1.320 (0.943–1.847)	0.105
70–79	1.751 (1.262–2.431)	0.001	1.643 (1.173–2.302)	0.004
≥ 80	2.832 (2.035–3.942)	< 0.001	2.022 (1.417–2.887)	< 0.001
Sex				
Male	1.000		**–**	
Female	1.068 (0.936–1.218)	0.331	**–**	**–**
Ethnicity				
White	1.000		**–**	
Black	1.041 (0.826–1.311)	0.736	**–**	**–**
Asia–Pacific	1.011 (0.830–1.231)	0.916	**–**	**–**
Other/Unknown	0.721 (0.374–1.392)	0.330	**–**	**–**
Marital status				
Single	1.000		1.000	
Married	0.915 (0.744–1.125)	0.397	0.826 (0.668–1.020)	0.076
Divorced	1.017 (0.741–1.396)	0.915	0.930 (0.674–1.282)	0.657
Widowed	1.304 (1.038–1.637)	0.023	0.852 (0.662–1.097)	0.214
Unknown	0.922 (0.597–1.424)	0.715	0.726 (0.463–1.138)	0.163
Tumor site				
Bilateral	1.000		**–**	
Unilateral	0.705 (0.264–1.885)	0.486	**–**	**–**
Tumor size (cm)				
Unknown	1.000		1.000	
≤ 2.0	0.590 (0.383–0.909)	0.017	0.790 (0.508–1.228)	0.294
2.1–4.0	0.660 (0.512–0.851)	0.001	0.606 (0.466–0.787)	< 0.001
> 4.0	0.849 (0.738–0.978)	0.023	0.882 (0.758–1.026)	0.103
Metastasis				
Localized	1.000		1.000	
Regional	1.011 (0.766–1.344)	0.938	1.210 (0.913–1.604)	0.185
Distant	1.867 (1.452–2.399)	< 0.001	2.037 (1.575–2.634)	< 0.001
Unknown	1.086 (0.660–1.787)	0.745	0.551 (0.331–0.918)	0.022
Surgery				
Non-surgical Therapy	1.000		1.000	
Subtotal/Near-total Thyroidectomy	0.595 (0.501–0.706)	< 0.001	0.573 (0.479–0.686)	< 0.001
Total Thyroidectomy	0.410 (0.347–0.484)	< 0.001	0.453 (0.380–0.540)	< 0.001
Radiation				
Refused/Unknown	1.000		1.000	
Yes	0.539 (0.473–0.614)	< 0.001	0.647 (0.558–0.751)	< 0.001
Chemotherapy				
No/Unknown	1.000		1.000	
Yes	0.494 (0.432–0.565)	< 0.001	0.581 (0.498–0.678)	< 0.001
Histopathology				
ATC	1.000		1.000	
PSCCTh	0.691 (0.542–0.880)	0.003	0.546 (0.425–0.701)	< 0.001

*HR*, hazard ratio; *CI*, confidence interval; *NSTh*, non-surgical therapy; *SNTTh*, subtotal/near-total thyroidectomy; *TTh*, total thyroidectomy

### Building and validating the nomogram

**Fig. 5:**
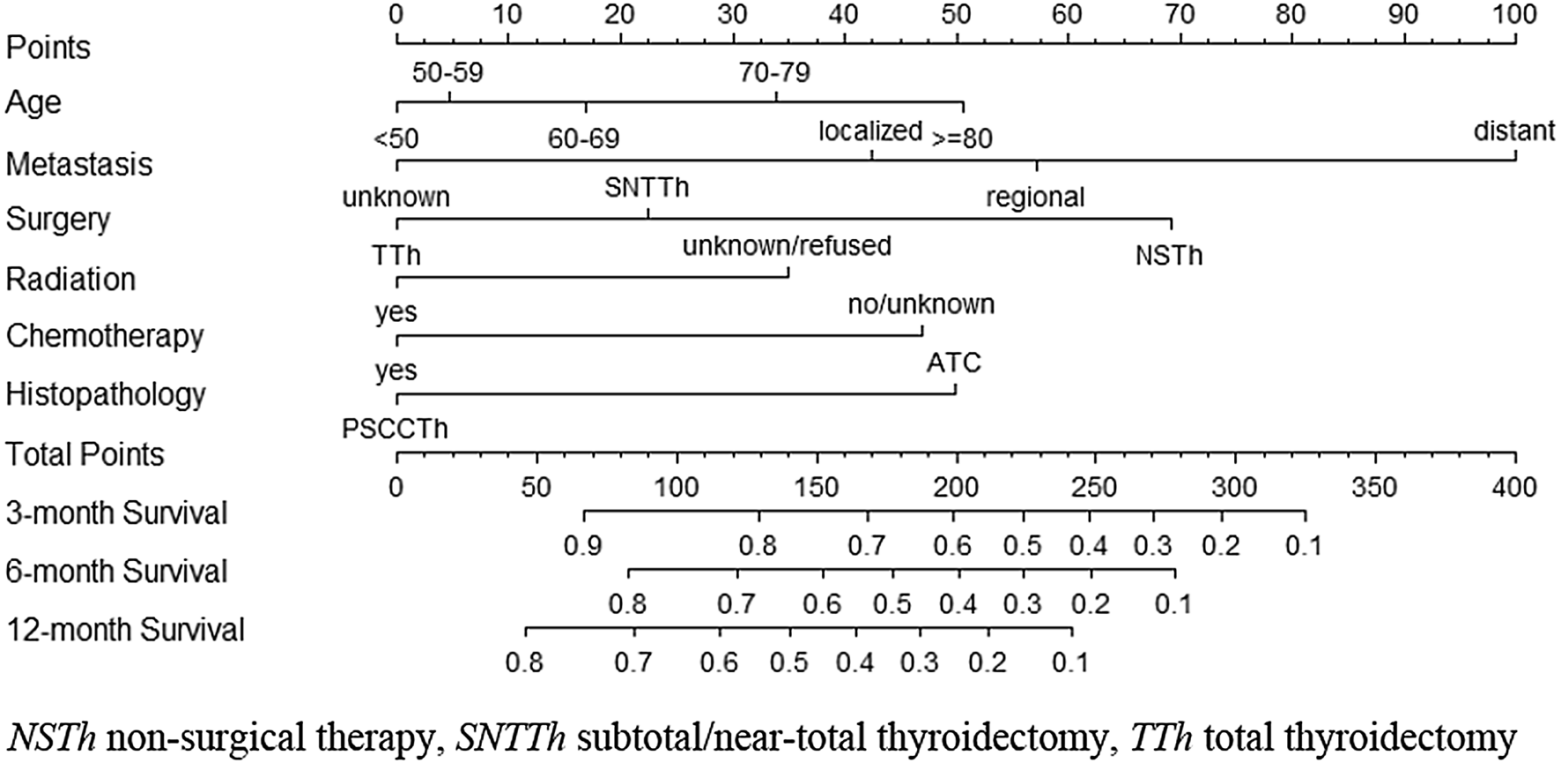
Nomogram for predicting 3-, 6-, 12-month OS of rATC patients in the training cohort

**Fig. 6:**
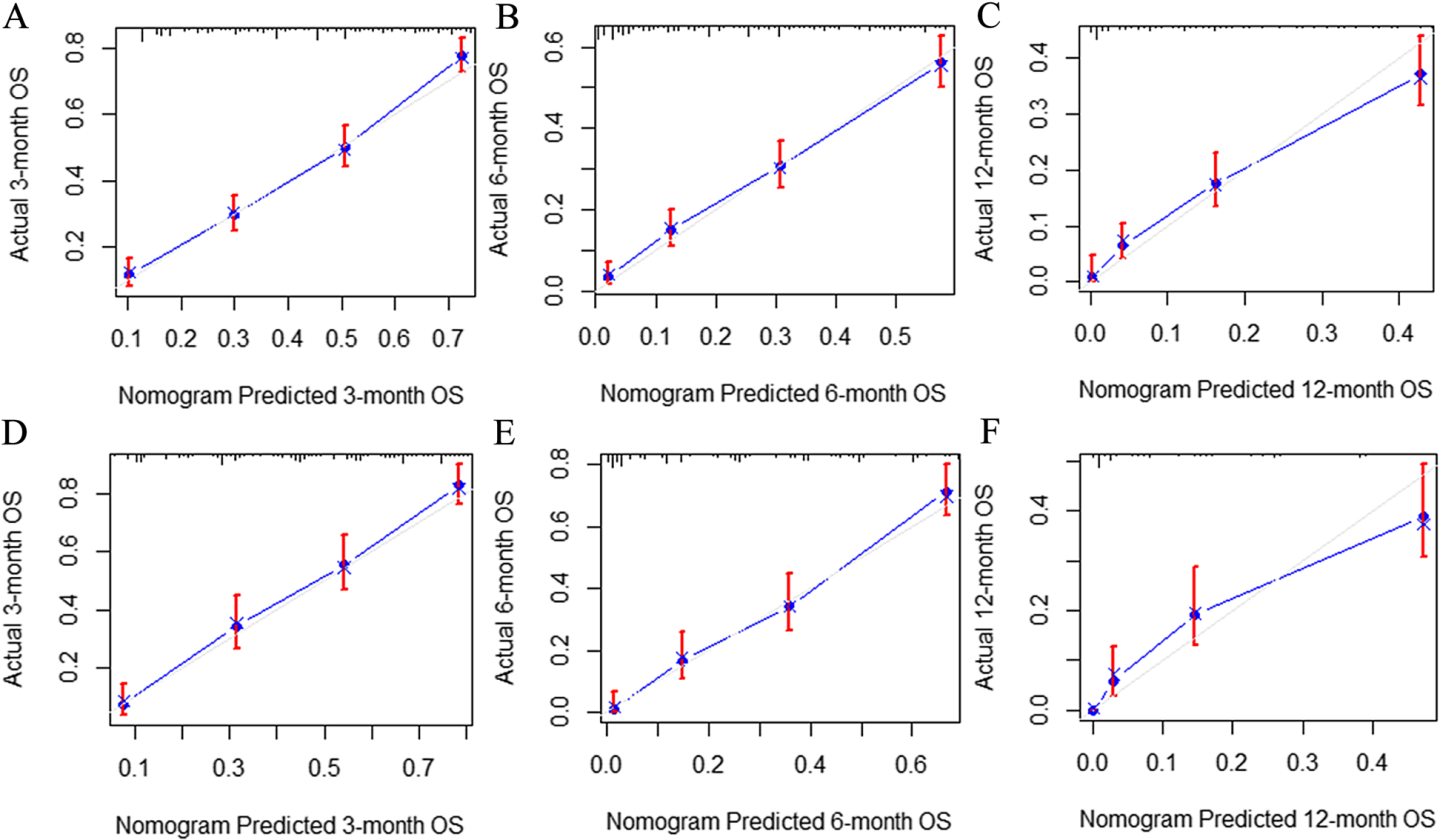
Calibration curves for training and validation cohort

The predictive model was virtually presented in the form of a nomogram (Fig. 5). The nomogram for predicting OS was constructed based on the following six independent risk factors: age (< 50, 50–59, 60–69, 70–79, and ≥ 80), metastasis (localized, regional, distant, and unknown), surgery (non-surgical therapy, subtotal/near-total thyroidectomy, total thyroidectomy), radiation (yes, refused /unknown), chemotherapy (yes, no/unknown), histopathology (ATC, PSCCTh). Each independent risk factor corresponds to a specific score by drawing a line straight upward to the points axis. The total points reflect the sum of the score of each factor and correspond to the prediction probability of the 3-, 6-, and 12-month OS by drawing straight down from the total points axis to the 3-, 6-, and 12-month survival axes. The C-index of the training cohort was 0.740 (95%: 0.721–0.759), and the validation cohort was 0.778 (95% CI 0.754–0.803), reflecting the good discrimination ability of the model. The calibration curves also showed good consistency in the probability of 3-, 6-, and 12-month OS between the actual observation and the nomogram prediction (Fig. 6a–f).

## Discussion

The *Fifth Edition of the WHO Classification of Endocrine and Neuroendocrine Tumors* refers to the Carl Linnaeus method, using the cellular origin as the basis for a new classification framework [5]. Tumor classification and subtyping are determined based on histological and molecular characteristics. Multiple lines of evidence indicate that SCCTh is the morphological pattern of ATC [3]. (1) In patients with pure squamous cell carcinoma, with or without components of differentiated thyroid cancer, approximately 87% show *BRAF*^*V600E*^ mutation, and their prognosis is similar to that of anaplastic thyroid cancer (ATC). (2) The probabilities of expressing *PAX8* and *TTF-1* in squamous cell carcinoma are 91% and 38%, respectively, confirming the origin from follicular cells. (3) In PSCCT cases, 3/4 of them are accompanied by differentiated thyroid cancer, mostly papillary carcinoma. (4) Pure squamous cell carcinoma without any components of differentiated thyroid carcinoma (i.e., meeting the definition of squamous cell carcinoma of the thyroid according to the 2017 WHO classification) carries *BRAF*^*V600E*^ mutation in approximately 60% of cases and has a prognosis similar to that of anaplastic thyroid carcinoma (ATC). Considering the above reasons, it is necessary to promptly conduct *BRAF*^*V600E*^ mutation testing for the diagnosis of PSCCT. The combined application of *BRAF* inhibitors (such as Dabrafenib, Vemurafenib, etc.) and *MEK* inhibitors (such as Trametinib, etc.) is effective for ATC or PSCCTh with *BRAF*^*V600E*^ mutation.

In this study, the median OS and median CSS of PSCCTh patients were higher than those of ATC patients, consistent with the results of Limberg's study [8]. The peak incidence of ATC and PSCCTh is concentrated in the age group of 60–79 years. Age is a common independent prognostic factor for ATC and PSCCTh patients. The CSS of ATC patients aged 60 or above is lower than that of patients under 60 years old, while the CSS of PSCCTh patients aged 80 or above is lower than that of patients under 80 years old. Surgery, radiotherapy, and chemotherapy are protective factors for CSS in patients with ATC, and related studies [9–12] have reached the same conclusion. Smallridge et al. [13] demonstrated that, for patients diagnosed with early ATC, surgical treatment, chemotherapy, radiotherapy, and systemic treatment can achieve optimal survival outcomes, while active palliative and clinical care are crucial for individuals with advanced ATC [14–17], but radiotherapy and chemotherapy are not prognostic factors for PSCCTh. Since PSCCTh is a rare type of thyroid cancer histology and clinical experience of this disease is limited, appropriate treatment for PSCCTh is controversial. Surgical treatment is a common independent prognostic factor for both ATC and PSCCTh patients, with a higher treatment rate for ATC compared to PSCCTh. Previous studies have indicated that surgical resection is associated with improved outcomes in patients with ATC [9–11]. Patients who undergo total thyroidectomy have a higher CSS compared to those who undergo subtotal or near-total thyroidectomy. Additionally, among patients who undergo total thyroidectomy, those with PSCCTh have a higher CSS rate than those with ATC. Surgery with a goal of an R_0_ resection and consideration of a lymphadenectomy should be the mainstay of therapy for these patients. Adjuvant therapies do not appear to impact overall survival when complete surgical resection is achieved, but may be beneficial after incomplete surgical resection or when surgery is unable to be performed [8, 18]. We have also found a higher proportion of ATC patients with distant metastasis. In rATC patients, age is an independent prognostic factor, with OS decreasing as age increases. Larger tumor diameter and distant metastasis are adverse independent prognostic factors that affect OS, possibly due to their strong invasiveness and the high burden or invasion of surrounding tissue and organs. Surgical treatment, especially total thyroidectomy, can effectively improve clinical symptoms and increase OS. Radiotherapy and chemotherapy are beneficial independent prognostic factors, but they are not independent prognostic factors for PSCCTh patients, which may be related to the small sample size of PSCCTh.

This study has some shortcomings. The limited number of patients with PSCCTh included in the study may have contributed to possible false-negative results. In addition, this was a retrospective study and some samples with incomplete data were removed, with only patients with complete information included, which may have led to bias.

## Conclusions

Although both ATC and PSCCTh are invasive thyroid malignancies, PSCCTh patients have a better prognosis in terms of CSS compared to ATC patients. We have established prognostic models for CSS in ATC and PSCCTh patients, but the prognostic factors for these two diseases are not entirely the same. This suggests that specific treatment and management plans need to be developed for these two types of thyroid cancer in clinical practice. We constructed a nomogram to provide a convenient and reliable tool for predicting OS in rATC patients, which would contribute to identifying high-risk patients for physicians.
